# Optimal duration of dual antiplatelet therapy after percutaneous coronary intervention or after acute coronary syndrome: Practical lessons from a review

**DOI:** 10.1007/s12471-017-1023-y

**Published:** 2017-07-31

**Authors:** J. M. ten Berg, B. Zwart, A. W. J. van ’t Hof, A. Liem, J. Waltenberger, R. J. de Winter, J. W. Jukema

**Affiliations:** 1 Department of Cardiology, St. Antonius Hospital, Nieuwegein, The Netherlands; 2 Department of Cardiology, Isala Klinieken Zwolle, Zwolle, The Netherlands; 3 Department of Cardiology, Franciscus Gasthuis, Rotterdam, The Netherlands; 4 Department of Cardiovascular Medicine, Universitätsklinikum Münster, Münster, Germany; 5 Department of Cardiology, Academic Medical Center, Amsterdam, The Netherlands; 6 Department of Cardiology, Leiden University Medical Center, Leiden, The Netherlands

**Keywords:** Acute coronary syndrome, Percutaneous coronary intervention, Dual antiplatelet therapy

## Abstract

To prevent recurrent ischaemic events, dual antiplatelet therapy (DAPT) is the standard of care after percutaneous coronary intervention and in the treatment of acute coronary syndrome. Recent evidence supports an adjusted DAPT duration in selected patients.

The current paper aims to encourage cardiologists to actively search for patients benefiting from either shorter or prolonged duration DAPT and proposes an algorithm to identify patients who are likely to benefit from such an alternative strategy.

Individualised DAPT duration should be considered in high-risk anatomic and/or clinical subgroups or in patients at increased haemorrhagic risk with low ischaemic risk. Both thrombotic and haemorrhagic risk should be assessed in all patients. In patients undergoing percutaneous coronary intervention, the interventional cardiologist could advise on the minimal duration of DAPT. However, in contrast to the minimum duration of DAPT for stent thrombosis prevention, longer duration DAPT is aimed at prevention of spontaneous myocardial infarction, and not at stent thrombosis, and thus the key to success is to treat the patient’s overall thrombotic risk.

The advice on the duration of DAPT must be documented in the patient’s records and communicated with the treating physician and general practitioner. DAPT duration should be reassessed at least on a yearly basis.

## Introduction

Dual antiplatelet therapy (DAPT) with acetylsalicylic acid in combination with a P2Y_12_ inhibitor is the standard of care after an acute coronary syndrome (ACS) and after percutaneous coronary intervention (PCI), to prevent recurrent ischaemic events such as stent thrombosis. DAPT is recommended with a class I level of evidence in the current guidelines [1, 2]. Clopidogrel is first choice in PCI patients with stable coronary artery disease, whereas the stronger P2Y12 inhibitors ticagrelor and prasugrel are mainly used in the ACS setting.

Restenosis rates have dropped dramatically since the introduction of drug-eluting stents (DES). However, first-generation DES were associated with a slight increase in stent thrombosis, which is the most feared complication of coronary stenting due to its very high mortality rates. Since the introduction of second-generation DES, together with the use of stronger P2Y12 inhibitors, rates of stent thrombosis have decreased by approximately 50% [3]. Second-generation DES are now considered superior in all aspects.

With improvement of the currently available stents, attention has shifted to the downsides of DAPT, such as the risk of bleeding. Premature discontinuation of clopidogrel has been shown to be the most important risk factor for stent thrombosis [4–7]. However, lower complication rates for coronary stents and increased awareness of bleeding on DAPT, led cardiologists to question the optimal duration of DAPT after coronary stenting.

From a mechanical point of view, DAPT is mandatory in the first months after stent implantation until endothelialisation has been completed. Interestingly, two recent studies using a new-generation DES demonstrated that DES was superior to BMS when combined with an ultra-short course (1 month) of DAPT [8, 9]. As the risk of late stent thrombosis is less of a concern with second-generation DES, prolonged DAPT is now more aimed at preventing (recurrent) myocardial infarction (MI) not related to the implanted stent. Thus, rather than focusing on the stent or the angiogram alone, the key to success is to treat the patient’s overall thrombotic risk.

In the last five years, multiple studies have been published comparing duration of DAPT after PCI and in ACS patients investigating either a shorter or prolonged DAPT regimen.

Although current ESC guidelines provide backup to individualise treatment, few patients in daily practice are currently being treated with a shorter or prolonged DAPT duration in our experience. This article summarises the results of relevant studies and meta-analysis and provides tools to individualise the duration of DAPT.

## Studies on optimal duration of DAPT

More than 10 randomised controlled trials (RCTs) and an almost equal number of meta-analyses on optimal DAPT duration after PCI have been performed. However, results are not easy to interpret, as length of treatment and comparison strategies varied (short versus standard DAPT, prolonged vs. standard DAPT and short vs. prolonged). Also, different definitions were used (e. g. bleeding definitions TIMI, BARC, STEEPLE, GUSTO).

### Shorter duration

In summary, the results of the 5 RCTs and several meta-analyses indicate that a shorter DAPT regimen (3–6 months) appears to be as effective as a standard DAPT duration (12 months) and might even provide a benefit in terms of bleeding. Of note, these studies included mostly low-risk patients [10–14]. Interestingly, a recent review found that 3 months of DAPT appears to be safe in patients with stable angina undergoing PCI, whereas in ACS patients, even though predominantly unstable angina/low risk, it was associated with increased ischaemic risk [15]. Currently, the REDUCE trial is addressing the issue of reduced DAPT duration in ACS patients. This physician-initiated, multicentre trial randomises 1,500 ACS patients treated with the COMBO stent to 3 vs. 12 months of DAPT [16].

### Prolonged dual antiplatelet therapy

The majority of RCTs after prolonged DAPT either failed to demonstrate a benefit of prolonged treatment or met the non-inferiority hypothesis. Two studies even suggested potential harm from prolonged DAPT in terms of increased rates of major bleeding events [17, 18]. Of note, none of the RCTs demonstrated an increase in fatal bleeding rates with prolonged DAPT, although the conclusions were limited by low event rates.

Although no consistent benefit of prolonged DAPT was observed in the individual RCTs for the ischaemic endpoints, most subsequent meta-analyses using pooled data did find a significant benefit of prolonged DAPT, but at the cost of increased bleeding events.

Consequently, it is now believed that DAPT continuation after 12 months reduces ischaemic events, but at the cost of increased bleeding rates. In the recent American College of Cardiology and American Heart (ACC/AHA) focused update on DAPT it was estimated that prolonged DAPT leads to an absolute decrease in stent thrombosis and ischaemic complications of ≈1 to 2% at the cost of an absolute increase in bleeding complications of ≈1% [2].

### Mortality in prolonged DAPT

Several previous studies have raised concerns that serious bleeding events resulting from prolonged DAPT might lead to increased rates of all-cause death, thereby offsetting the reduction in cardiac death and nonfatal ischaemic events. This suspicion arose in the DAPT study and in some [19–22], but not all, meta-analyses [23, 24]. The DAPT study [25] was by far the largest trial demonstrated a benefit of prolonged DAPT (30 vs. 12 months) in reducing stent thrombosis (0.4 vs 1.4%, *p* = 0.001) and major adverse cardiac and cerebrovascular events (4.3 vs. 5.9%, *p* = <0.001), but at the cost of significantly more GUSTO (global utilisation of streptokinase and t‑pa for occluded coronary arteries) moderate or severe bleeding (2.6 vs. 1.6%, *p* = 0.001). An unexpected, but important finding was a borderline significant (*p* = 0.05) excess mortality (all-cause death 2.0% in prolonged DAPT vs. 1.5% in placebo-treated patients) due to more non-cardiovascular deaths [25, 26].

The meta-analysis in the ACC/AHA focused update addressed this topic and found “weak evidence” of increased mortality with prolonged DAPT in those RCTs that successfully achieved their predefined enrolment target [3]. Another analysis in this review suggested increased mortality with prolonged DAPT in patients without prior history of ACS but not in patients with a history of prior ACS.

### Prolonged DAPT after MI

Most notably, patients who have had an MI are deemed at risk for recurrent ischaemic events. Unfortunately, few studies focused on this specific patient group.

The PEGASUS is a double-blind RCT including 21,162 patients with a previous myocardial infarction and additional ischaemic risk factors [27]. In patients treated with prolonged DAPT with ticagrelor an absolute risk reduction of cardiovascular death, myocardial infarction and stroke of 1.3% at the cost of an equally increased risk of major bleeding events was found. All-cause death did not differ significantly.

Udell et al. analysed in a recent meta-analysis in patients with previous MI either treated medically or managed with PCI, whether prolonged DAPT after one year (mean difference in achieved duration of DAPT 30 months) is beneficial. They found a reduction in major adverse cardiovascular events including stent thrombosis (6.4 vs. 7.5%; risk ratio, RR 0.78, *p* = 0.001) and cardiovascular death (2.3 vs. 2.6%; RR 0.85, *p* = 0.03), but no significant effect on overall mortality (RR of 0.92 (95% CI 0.83–1.03; *p* = 0.13)) [24]. An increased rate of major bleeding events was observed (1.85 vs. 1.09%; RR 1.73, *p* = 0.004), but not of fatal bleeding events.

Overall, it is demonstrated that the benefit of prolonged DAPT in reducing future ischaemic events is much stronger after MI as compared to in stable coronary artery disease (SCAD) patients [28].

## Interpretation

Conceptually, some of the foregoing conclusions are difficult to interpret. How can shorter-duration DAPT be as effective as the ‘standard regimen’, when on the other hand extended-duration DAPT reduces recurrent ischaemic events, including stent thrombosis?

Moreover, the paradigm that platelet inhibitors reduce thrombotic risk, but increase bleeding, in our opinion still holds true for the various strategies. As this principle was observed in studies after prolonged DAPT duration, but not in studies after shorter duration, this observed asymmetrical treatment effect might be due to the low event rates, patient selection or chance.

Indeed, it proves useful to look in detail at these studies. With regard to stent thrombosis, an important endpoint, all 5 studies investigating a shorter DAPT duration are hampered by very low (<50% of expected) event rates with stent thrombosis rates varying from 1 to 6 per treatment arm. Therefore, the outcomes regarding stent thrombosis should be interpreted with caution.

With regard to prolonged DAPT, it is important to acknowledge that in most trials patients were randomised after the first year and only if they did not have any major bleeding events. On the other hand, this reflects clinical practice, in which the decision to extend DAPT after a year can be revised at any stage should haemorrhagic or ischaemic events occur.

Because both ischaemic outcomes and bleeding events are reported in most studies, in some studies even as a composite endpoint, it is difficult to know how to weigh haemorrhagic risks against ischaemia risks. The impact of bleeding events should, however, not be underestimated. It is a strong predictor of mortality; in some studies even stronger than myocardial infarction [23, 29].

The ADAPT-DES study provided important detailed information on this subject [30]. The authors demonstrated that stent thrombosis, although infrequent with 0.9%, was associated with very high mortality rates ranging from 15 to 38% (the latter for early stent thrombosis). Spontaneous, not stent thrombosis related MI was less frequently fatal (mortality rates ranging from 0.8–7.5%). Mortality rates after clinically relevant bleeding events were comparable with spontaneous MI. In conclusion, as there is evidence of potential harm associated with prolonged DAPT, it should only be considered in carefully selected patients at substantially high ischaemic and low haemorrhagic risk.

### Current guidelines

The 2014 European Society of Cardiology (ESC) guideline on myocardial revascularisation advises treating patients with SCAD with DAPT for 6 months after DES implantation (class I level B) [1]. Shorter DAPT duration may be considered in patients with a high haemorrhagic risk (IIb, A), whereas the guideline advises that DAPT may be used for more than 6 months in patients at high ischaemic and low haemorrhagic risk (IIb, C). The 2015 ESC guideline on non-ST-segment elevation ACS recommends considering a shorter DAPT duration of 3–6 months after DES implantation in patients deemed at a high haemorrhagic risk (IIb, A) [31]. This is remarkable, because evidence is based on studies that included predominantly low-risk patients with SCAD. Furthermore, the guideline includes a IIb (level A) recommendation for continuing DAPT beyond 1 year in selected patients with ACS ‘after careful assessment of the ischaemic and haemorrhagic risks of the patient’. However, it does not specify how we can identify these patients.

The ACC/AHA Focused Update provides an excellent overview of the relevant studies. However, the recommendations are essentially not very different from current guidelines and again lack specific guidance on identifying the patients suitable for individualised treatment [2, 3]. In ACS patients, either prolonged or shorter DAPT ‘may be reasonable’, (IIB) depending on the presence or absence of high haemorrhagic risk. Importantly, the authors consider this regardless of treatment strategy.

For patients with SCAD, the guideline recommends continuing DAPT in DES-treated patients after 6 months if there is ‘no high risk of bleeding and no significant overt bleeding on DAPT’. Interestingly, ischaemic risk is not considered. In our interpretation of the currently available studies, however, evidence for prolonged DAPT, particularly after one year, in patients with SCAD is weak, especially in the absence of previous MI. Prolonged DAPT may even cause harm.

## DAPT duration: the right strategy for the right patient

In conclusion, the aforementioned studies and reviews suggest that a minimum duration of six or even three months is effective in low-risk patients with SCAD after second-generation DES implantation, whereas in ACS patients, extension of DAPT beyond 12 months appears to be beneficial only in selected subgroups, most notably patients with prior MI.

The large RCTs and high quality meta-analyses do, however, not support *one* new standard DAPT duration in all patients. They rather support a personalised treatment in which the duration of DAPT is determined on an individual patient basis, reflecting an accurate trade-off between the individual ischaemic and haemorrhagic risks.

### Use of risk scores

Risk scores may aid in this decision-making. Previously, several clinical patient characteristics (e. g. ACS, diabetes, impaired left ventricular function), as well as procedural (e. g. dissection, bifurcation stenting) and angiographic factors (e. g. undersizing of the stent, small stent diameter) have been identified as risk factors for stent thrombosis [4, 32, 33]. As demonstrated in the DAPT study, however, approximately 50% of recurrent atherothrombotic events are not related to stent thrombosis. Hence, the patient’s overall ischaemic risk should be considered when it comes to longer duration of DAPT.

Traditionally used scores to assess ischaemic risk include the TIMI and GRACE risk score [34, 35]. While these risk scores are very useful in the initial assessment of ischaemic risk and hence in guiding treatment and timing of coronary angiography, the clinical applicability in the outpatient setting is limited because of the parameters used.

Recently, the DAPT risk score was published [32]. Although the DAPT score helps us to identify patients who are likely to benefit from or who are likely to be harmed by prolonged DAPT, it must be noted that this score has yet to be validated prospectively.

Bleeding risk can also be assessed by commonly used risk scores such as the CRUSADE and the HAS-BLED scores [36, 37]. However, the applicability of these risk scores is also limited. The CRUSADE score was designed for in-hospital use in patients with non-ST-segment-elevation myocardial infarction, whereas the HAS-BLED score is used in patients with atrial fibrillation. Furthermore, it must be noted that the predictive value of the HAS-BLED score is limited. The recently updated ESC atrial fibrillation guideline acknowledges that the HAS-BLED score is merely a tool to identify modifiable risk factors [38].

Another difficulty in clinical daily practice is that some risk factors are associated with both haemeorrhagic and ischaemic risk (e. g. renal function, age). Interestingly, the DAPT study found that higher age increased haemorrhagic risk more than ischaemic risk [32].

### Selecting the right patient for the right therapy

So here we are. Decision-making for DAPT duration has never been so complex, but it is clear that a one-size-fits-all approach is outdated. However, we currently lack a comprehensive risk score to identify ischaemic and haemorrhagic risk in patients with coronary artery disease. The DAPT score might prove a very useful tool for prolonged DAPT but its validity has yet to be proven prospectively. Other existing risk scores focus more on the patient’s initial risk rather than on the long-term risk. Although we encourage identification and documentation of the patient’s risk with these risk scores, a different approach is needed to establish the best strategy in individual patients.

Therefore, we propose to start individualised therapy with a few well-defined and recognisable patient groups as set out below. Although this is a simplification of risks, we believe that there is sufficient evidence to individualise treatment duration in these patients on the two sides of the spectrum. Tables 1, 2 and 3 describe the three patient groups that are likely to benefit from an alternative strategy.

**Table 1:** Proposed treatment algorithm for reduced DAPT duration

Shorter DAPT duration (3 months in stable CAD; 6 months after ACS)
Single-vessel PCI
And
One of the following criteria for high bleeding risk: – Prior intracranial haemorrhage – Recent major bleeding – (N)OAC long-term therapy – Frailty and age >75 years – Malignancy

*DAPT* dual antiplatelet therapy, *CAD* coronary artery disease, *ACS* acute coronary syndrome, *PCI* percutaneous coronary intervention, *(N)OAC* (Non-vitamin-K) oral anticoagulant

**Table 2:** Proposed treatment algorithm for ultra-short DAPT duration

Ultra-short DAPT duration (1 month)
Single-vessel PCI for stable CAD
*And*
Use of latest generation stent with CE approval for 1 month of DAPT use (e. g. Biofreedom or Endeavor)
*And*
Patient is deemed to be at *very* high bleeding risk^a^ **or** scheduled for urgent surgery

*DAPT* dual antiplatelet therapy, *CAD* coronary artery disease, *CE* Conformité Européenne

^a^Very high bleeding risk: risk factors mentioned in Table 1 and additional risk factors at physician’s discretion

**Table 3:** Proposed treatment algorithm for prolonged DAPT duration

Prolonged DAPT duration
Presentation as ACS or previous history of ACS; regardless of treatment strategy
And
No major bleeding events during the first course of DAPT and no long-term (*N*)OAC
And
One of the following additional criteria for high thrombotic risk: – prior stent thrombosis – recurrent MI – diabetes mellitus – peripheral artery disease – multivessel coronary disease – complex coronary stenting^a^

*DAPT* dual antiplatelet therapy, *ACS* acute coronary syndrome, *MI* myocardial infarction,* (N)OAC* (Non-vitamin-K) oral anticoagulant

^a^Left main stent, bifurcation stenting, long or overlapping stents, last remaining vessel, venous graft percutaneous coronary intervention

The algorithms are based on the standard treatment regimen according to current ESC guidelines, i. e. 6 months of DAPT after elective PCI for SCAD or 12 months after ACS. Shorter duration is defined as three months in SCAD (or 1 month if an ultrashort duration is indicated) or six months after ACS. Prolonged DAPT can be continued for multiple years (in line with the DAPT study and the PEGASUS study), provided that this strategy is evaluated annually and amended if adverse events, such as bleeding events, are encountered. There are currently no data to support continuation of DAPT after three years.

We have sought to describe clear and undisputed subgroups. We only included risk factors which have repeatedly been shown to be risk factors for either haemorrhagic or recurrent ischaemic events [2, 4, 28, 32, 39, 40]. However, it is important to emphasise that this is not a final or static algorithm. Its aim is to identify, on both sides of the spectrum, patient groups that are likely to benefit from an alternative strategy. Patients not fitting the descriptions in the tables – or meeting criteria compatible with both high hameorrhagic and thrombotic risk – should be treated with standard DAPT duration. On the other hand, we obviously encourage alternative strategies in individual patients when this is motivated and documented.

To illustrate decision-making in clinical practice we have included two hypothetical cases.

#### Case 1: high ischaemic risk, prolonged dual antiplatelet therapy (DAPT)

A 52-year-old man, known diabetic and smoker, is admitted with an anterior ST-segment-elevation myocardial infarction (STEMI) for which he undergoes successful primary percutaneous coronary intervention (PCI) with implantation of two stents. There is diffuse moderate disease in the right and circumflex coronary artery for which no intervention is needed at present. The angiogram of his femoral artery taken at the end of the procedure also showed peripheral artery disease. There is no bleeding history. The interventional cardiologist notes in his PCI report that this patient might be suitable for prolonged DAPT.

He is seen at the clinic for follow-up at three months and again at one year and remained symptom free from a cardiac point of view. There are no bleeding events during the first year. His cardiologist discusses the considerations for prolonged DAPT with him and they decide to continue the DAPT. He will come back after one year and if the DAPT is well tolerated, it might be reasonable to continue DAPT for even longer.

- Ischaemic risk: High (multivessel disease), diabetes, peripheral artery disease
- Haemorrhagic risk: Low (no bleeding history, relatively young age)

#### Case 2: high haemorrhagic risk, shorter dual antiplatelet therapy (DAPT)

A 67-year-old woman with osteoporosis and hypertension presents to the outpatients’ clinic with a 6-month history of angina. Coronary angiography demonstrated a narrowing in the mid-portion of the right coronary artery. She subsequently underwent a percutaneous coronary intervention during which a drug-eluting stent is implanted, with excellent result. There is only minor disease in the left coronary artery. Three months later, she is admitted with a gastro-intestinal bleeding with a three point drop in haemoglobin. Colonoscopy and computed tomography demonstrate a T2N0M0 tumour for which she will need surgery. The surgeon discusses the discontinuation of DAPT with her cardiologist and in light of the low ischaemic risk and the high haemorrhagic risk they agree to discontinue the P2Y_12_ inhibitor. Aspirin should be continued.

- Ischaemic risk: Low (PCI for stable CAD, no other high-risk ischaemic features)
- Haemorrhagic risk: High (recent gastro-intestinal bleeding; active malignancy)

### Future perspective and implementation in daily practice

Algorithms like the DAPT-scores should be tested in future prospective studies to prove their validity and applicability. When we test such strategies, it is important to acknowledge a distinction according to clinical presentation: SCAD or ACS patients.

We believe in an important advisory role for the interventional cardiologist. The interventional cardiologist who performs the PCI, knows the angiographic complexity and results of the procedure and is therefore best equipped to determine the *minimum* DAPT duration to be incorporated in the PCI report. For patients who are conservatively managed, the treating physician is in the lead.

In all scenario’s, it is essential that the recommended DAPT duration and preferred P2Y12 inhibitor are included in the discharge letter. This advice should be communicated to the referring district hospitals, if applicable, and to the patient’s general practitioner.

Obviously, DAPT duration is not a static advice and it can be revised at any time during the patient’s follow-up (e. g. recurrent myocardial infarct, stroke or bleeding complications). This implies that there is an important role for the cardiologists who provide clinical follow-up for their patient in clinic. At least, the DAPT duration should be rediscussed at the time of the first follow-up visit after discharge and at every annual follow-up.

With this paper, we intend to increase awareness and emphasise the importance of an individualised treatment strategy in patients undergoing PCI or presenting with ACS, regardless of treatment strategy.

We hope to encourage cardiologists in the Netherlands and abroad to join us in this strategy. The ultimate goal is to offer a personalised DAPT recommendation to any patient suffering from ACS or undergoing PCI. Hopefully, with the help of and in cooperation with the Netherlands Society for Cardiology (NVVC) and its working groups, we will be able to implement personalised DAPT duration in the near future.
